# Mysticism in the courtroom in 19th-century Europe

**DOI:** 10.1177/0952695118761499

**Published:** 2018-03-26

**Authors:** Andrea Graus

**Affiliations:** University of Antwerp, Belgium

**Keywords:** Catholicism, criminal justice, expert testimony, legal history, supernatural

## Abstract

This article examines how and why criminal proceedings were brought against alleged cases of Catholic mysticism in several European countries during modernity. In particular, it explores how criminal charges were derived from mystical experiences and shows how these charges were examined inside the courtroom. To bring a lawsuit against supposed mystics, justice systems had to reduce their mysticism to ‘facts’ or actions involving a breach of the law, usually fraud. Such accusations were not the main reason why alleged mystics were taken to court, however. Focusing on three representative examples, in Spain, France and Germany, I argue that ‘mystic trials’ had more to do with specific conflicts between the defendant and the ecclesiastical or secular authorities than with public concern regarding pretence of the supernatural. Criminal courts in Europe approached such cases in a similar way. Just as in ecclesiastical inquiries, during the trials, judges called upon expert testimony to debunk the allegedly supernatural. Once a mystic entered the courtroom, his or her reputation was profoundly affected. Criminal lawsuits had a certain ‘demystifying power’ and were effective in stifling the fervour surrounding the alleged mystics. All in all, mystic trials offer a rich example of the ways in which modern criminal justice dealt with increasing enthusiasm for the supernatural during the 19th century.

## Introduction

Why did 19th-century criminal justice take an interest in cases of stigmata, prophecies and other mystical phenomena? What criminal charges were filed against alleged mystics and how were they examined in the courtroom? What public concerns did these trials raise? This article revolves around these questions by exploring how three women from different European territories, who claimed to be Catholic mystics, were brought to court. The early modern period offers rich examples of the role of criminal justice in the evaluation of the supernatural, especially in the form of witch trials (see, e.g. Ankarloo, Clark and Monter, 2002; Duni, 2016; Stokes, 2011). During later modernity, however, we find fewer instances of the ways in which secular courts judged crimes with supernatural or occult implications. Such instances include trials of fraud in spirit photography (Monroe, 2003), the imprisonment of charismatic leaders of sects and satanic societies (Ziegler, 2012), the conviction of ‘criminal telepaths’ and fortune-tellers (Wolffram, 2009), and the legal pursuit of magnetizers and lay healers (Lafferton, 2007; Wolffram, 2017).

The cases cited fell outside the boundaries of established religion and, perhaps for this reason, appeared mainly to be under the responsibility of the civil powers. It is thus clear that modern criminal justice was concerned with deviant religious cults and the lucrative exploitation of superstition. This article, however, deals with Christian mysticism: a matter traditionally policed by the Church and where the intervention of the law was often deemed intrusive. Historically, mysticism has implied a dialectical dynamic between the contemplative and the transformative (Katz, 1983; Sheldrake, 2003). According to Michel de Certeau, mystics are found on the margins of religion and society, embodying a subversive ‘otherness’ that challenges the establishment (Bocken, 2013; De Certeau, 1982). For modern criminal courts to intervene in cases of mysticism, they had to provide powerful justification for their involvement in legal terms. Accusations of fraud were usually behind lawsuits against alleged mystics; but there were also other implicit reasons – embedded within the political and religious conflicts of the ‘culture wars’ in 19th-century Europe – for the cases to make it to court. Most lawsuits had more to do with the particular agendas of civil and ecclesiastical authorities than with public concern regarding fraud. By exploring the politico-religious meaning of mystic trials, this article joins other scholars in analysing the political function of Christian mysticism in European modernity (see, e.g. Blackbourn, 1993; Multon, 2003; Van Osselaer, forthcoming).

Bringing cases of supposed mysticism before the civil courts was not common in the 19th century. Research has revealed no more than a dozen cases across France, Spain, Italy, Belgium, Germany and Britain. This article deals with two of the most renowned cases in France and Germany, and with the only apparent case occurring in Spain, but where the scandal was such that it became known across Europe. Alongside Spain, the number of mystics brought to court in Italy in the 19th century is almost non-existent. A partial explanation of this fact is that, in these Catholic countries especially, the juridical setting of the early modern inquisition, based on testimony and evidence, already proved to be unfit for cases of mysticism, which appeared to be more suited to theological inquiry (Shuger, 2015). This idea probably persisted during modernity, relegating such cases to traditional diocesan investigation, hindering the intervention of the legal system.

In this article, I call those who claimed to experience phenomena related to Christian spirituality ‘mystics’. Their mystical experiences, such as ecstasy, were sometimes accompanied by alleged miracles such as stigmata. The trials examined the claims relating to both. We must take into account, however, that, once brought into the courtroom, mystics were generally considered to be ‘self-styled’ and not part of the accepted Christian tradition. When confronted with alleged cases of mysticism, 19th-century criminal justice looked for evidence of fraud and not for evidence of the supernatural. Nevertheless, during both pretrial investigations and the trial itself, debates concerning how ecstasy or stigmata could be feigned raised public concern about the authenticity of miracles and mystical phenomena. As I aim to show, despite the courts’ intentions, elements of the press and the population perceived these proceedings as trials of the supernatural.

The first section of the article explores the public threats caused – among other events – by a Catholic revival in 19th-century Europe, which forced the intervention of the law in matters traditionally policed by the Church. The cases of Sor Patrocino, Rosette Tamisier and Catherine Filljung will be discussed; three Catholic mystic women brought before the court in Spain, France and Germany, respectively. In the following sections, these cases will be used as the basis for an explanation of typical strategies employed by criminal courts in Europe, attempting to bring before the law alleged Catholic mystics and prosecute them. Despite the national differences, we find similar legal procedures when confronting such mystics. Shared features range from the juridical mechanisms that transformed mystical phenomena into criminal facts, to the involvement of expert testimony, such as psychiatrists, during the trial. By demonstrating the political, juridical and medical assumptions apparent in mystic trials, this article aims to contribute to scholarship in cultural and religious history, and to the history of science, exploring the intersection between modern criminal justice and the supernatural (see, e.g. Edelman, 1995: ch. 1; Monroe, 2008: ch. 4; Wolffram, 2009: ch. 5).

## Mysticism: A case for criminal law?

Europe witnessed a revival of Catholic mysticism during the ‘culture wars’ – or the secular-religious conflicts of the 19th-century – during which the Catholic Church struggled with anticlericalism and the establishment of liberal governments (Clark and Kaiser, 2003). From Napoleonic Italy to Republican France and Bismarckian Germany, the political turmoil became fertile ground for an ‘ideological’ manifestation of the supernatural. Peasant women and children were the main actors in this Catholic revival, which was notably manifest in the form of Marian apparitions laden with political meaning, and through apocalyptic prophecies about the triumph of the Pope and the restoration of the *ancien régime* (see, e.g. Blackbourn, 1993; Bouflet and Boutry, 1997).

In relation to this scenario, Catholic mystics became symbols that were used in support of politico-religious causes. In Italy, lay mystics such as Anna Maria Taigi and Palma Matarelli prophesized the election of Pius IX and the devastating consequences of the culture wars for Catholics (MacLaren, 2012; Multon, 2003). In Belgium and Germany, the stigmatic Louise Lateau became emblematic in opposing the *Kulturkampf* (Van Osselaer, forthcoming). In France, at the beginning of the Third Republic, Berguille Bergadieu and Marie-Julie Jahenny announced the return of the monarchy and the rise of the Count of Chambord (Henri V), Legitimist pretender to the throne. The millenarist and messianic tone of their revelations pleased ultramontane aristocrats who had lost their privileges after the French Revolution (Kselman, 1983).

The Catholic revival coincided with heated debates concerning modern and gospel miracles, fostered, on the one hand, by a positivist approach to the supernatural, and, on the other, by a renewal of Biblical studies. With regard to the latter, the German-Protestant theologian David Friedrich Strauss fuelled the controversy with his book, *Das Leben Jesu* [The Life of Jesus] (1835), in which he disagreed with rationalist and supernaturalist approaches to gospel miracles and argued for the mythological basis of the New Testament (Craig, 1986; Gregory, 1992). Regarding modern miracles, the 19th-century is renowned for its positivist and medicalized approach to allegedly supernatural phenomena. The Salpêtrière School, which sympathized with the anticlerical agenda of the Third Republic, championed the pathologization of religious experiences (Didi-Huberman, 2003; Goldstein, 1982). Cases of alleged demonic possession and miraculous healing, such as the possessed of Morzine and the Lourdes *miraculées*, were integrated into the discourse on hysteria (Carroy, 1981; Edelman, 2003; Harris, 1999). In the fight against this approach, we find Catholic doctors such as Antoine Imbert-Gourbeyre, a French Legitimist, who attempted to prove the supernatural origin of stigmata, ecstasy and the Lourdes miracles in a book that challenged the ‘free-thinkers’ of the Salpêtrière (Imbert-Gourbeyre, 1894; Sandoni, 2014). The acknowledgement or debunking of miracles was thus part of the politico-religious clashes of the culture wars.

Other 19th-century approaches to the supernatural included those fostered by new religious movements such as Victorian Spiritualism and Allan Kardec’s Spiritism, which emerged from the fashion for ‘turning-tables’ and spirit communication in the 1850s. Such movements aimed not only to transform Christian spirituality but science and society as well; for example, presenting séances as scientific proof of survival after death and advocating utopian socialism and women’s emancipation (Hayward, 2007; Mülberger, 2016; Sharp, 2006). Female mediums across Europe became feminist activists, spreading their ideals through ‘trance texts’ against Catholicism and scientific materialism (Owen, 2004). Psychiatrists dispossessed mediums of their experiences, as they had done with the mystics, medicalizing their trances using theories of hysteria, automatism and hallucination (see, e.g. Edelman, 2003; Le Maléfan, 1999).

Thus, it is clear that the 19th-century uses of the supernatural implied an ideological intent and constituted a political threat within the European secular–Catholic conflicts. What was the role of modern justice in this respect, especially when the threat came from Catholic mystics; that is, individuals whose repression and control had traditionally been undertaken by the Church? To understand the role of the legal system, it is useful to look briefly at how the Catholic Church and the courts interacted in Europe before the 19th century.

Since the early Middle Ages, the Church had accepted the intervention of judicial powers in cases of repeated or major heresy, where the defendant had to follow a *judicium Dei* or trial by ordeal. With the abandonment of ordeal after the Fourth Lateran Council (1215), most European countries and the Catholic Church adopted an inquisitorial system based on the Roman canon law of evidence. This system of proof primarily relied on testimony and aimed to find ‘objective’ evidence of the facts, such as eyewitnesses. By law, torture could be used to force a confession from the defendant (Langbein, 1977). An official investigator conducted an extensive pretrial inquiry and interviewed witnesses in secret. As in canonization causes, a dossier with the written testimony was created and presented to the judges (Vidal, 2007). England was the only country using trial by jury at that time. Enlightenment thinkers criticized the use of torture and the secrecy of the inquisitorial procedure and, in 1791, France introduced the jury into criminal justice (Donovan, 2010; Johnson, Wolfe and Jones, 2008).

During the Enlightenment, and especially after the French Revolution, many thinkers advocated secularizing the state. They thought that offences against religion or against morality should not be punished by law (see, e.g. Montesquieu, 1995[1748]). According to Cesare Beccaria, an influential Italian jurist, offences should only be judged with regard to the damage caused to public peace and safety (Carbasse, 2014). While small fines or police warnings against ‘troublesome’ mystics became increasingly common (see, e.g. Canioni, 2014), bringing them before a court was an extreme measure reserved for cases having a powerful impact on public opinion or disturbing public order. A common feature shared by the Christian mystics who were taken to court during the 19th-century was that their cases had moved beyond an ‘acceptable’ level of religious enthusiasm, arousing the interest of the press and causing a public disturbance. This included inconvenient events such as uncontrolled gatherings at sites where ‘miracles’ had taken place, roads becoming unusable due to crowds of pilgrims, illegal commerce in alleged holy objects, indiscriminate publicity of the phenomena and the spread of political prophecies. In such cases, justice systems treated the events as a threat to public order and targeted the source of the disturbance, whether it was the prophet of a growing sect – such as Eugène Vintras (1807–1875), who was prosecuted (Garçon, 1928) – or the alleged mystics who will be introduced below. For example, in Germany, Catherine Filljung was declared a ‘danger to public safety’ for obtaining money from ‘credulous people’ while posing as a martyr (Pelt, 1934: 236–7).

**Figure 1.**
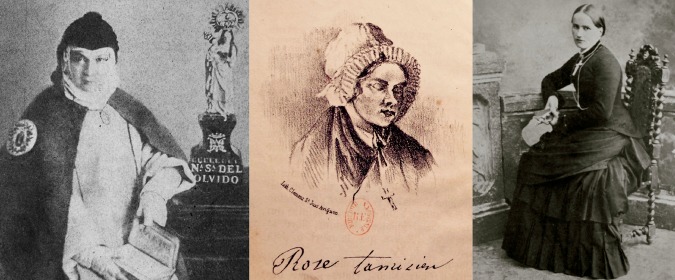
(a) Sor Patrocinio (Retrato de María Rafaela Quiroga. Sor Patrocinio, IH/7576/3, courtesy of Biblioteca Nacional de España); (b) Rosette Tamisier (Garçon (1929), *Rosette Tamisier ou la miraculeuse aventure*. Paris: L’Artisan du Livre); and (c) Catherine Filljung (Ebel (1932), *Soeur Catherine.* Paris: Téqui).

In the following, I sketch the cases of the Franciscan nun Sor Patrocinio (Madrid), and the French laywomen Rosette Tamisier (Provence) and Catherine Filljung (Lorraine) (Figure 1). These examples, which I will refer to throughout the sections below, allow the exploration of common features of mystic trials in 19th-century Spain, France and Germany – with Filljung’s case occurring during the annexation of Alsace-Lorraine to the German Empire and thus falling under German jurisdiction. As mentioned above, these kinds of cases were quite exceptional. While for the majority there are only brief references and no additional sources to expand the subject matter, for the cases presented here there is rich archival material and a wide range of printed primary sources: from books and transcriptions of court hearings to press articles and caricatures. The fact that these reports sometimes originated abroad indicates the significance of these trials at the time.

### Sor Patrocinio

In the late 1820s, Sor Patrocinio (1811–1891), a Spanish Franciscan nun who displayed stigmata, became renowned as ‘the nun with the wounds’ (see, e.g. Jarnés, 1929). During the First Carlist War (1833–1839), in which absolutists (Carlist) confronted liberal factions over the succession of the Spanish throne, Sor Patrocinio reported visions featuring the rise of the absolutist pretender (Dos Amigos Filósofos, 1868). The liberal governor of Madrid could not tolerate the resulting turmoil and filed a lawsuit against ‘the nun with the wounds’. She was accused of faking the stigmata and of attempting to subvert the state with her prophecies. The newspapers covered the judicial proceedings and Sor Patrocinio became a target of the republican and anticlerical press (Graus, forthcoming). After the trial, the nun was condemned to forced removal from her convent and placement in another, far from the Royal Court (Voltes, 1994). Over time, however, Sor Patrocinio regained the trust of the monarchy and became spiritual advisor to the queen. Her trial exemplifies how, in times of crisis, mystical phenomena could be transformed into political crime. The scandal was well known and reported in other European countries (see, e.g. Fonvielle, 1872).

### Rosette Tamisier

If Sor Patrocinio’s case symbolizes the ideological clash between liberalism and absolutism in Spain, Tamisier’s represents a particular battle in the ‘longer-running *guerre des deux Frances*’ (McMillan, 2003: 77). The ‘two Frances’ of the time were the Republican, which was secular and anticlerical, and the Catholic ultramontane (Seippel, 1905). In the early 1850s, Rosette Tamisier (1818–1899), a peasant woman from Provence, claimed to experience mystical phenomena such as miraculous Communion. She became renowned, however, for one particular ‘miracle’. One day, as she was praying before a painting of the pietà in the church of Saint Saturnin, the wounds of Christ began to ‘bleed’. Despite being discredited by the Archbishopric of Avignon, the phenomenon attracted thousands of pilgrims to the village. The public prosecutor decided to intervene and Tamisier was accused of showing contempt for a cult object. The Catholic and Republican press covered the juridical proceeding and transcribed the court hearings in the newspapers (see, e.g. ‘Cours et tribunaux’, 1851). Popular enthusiasm faded once Tamisier was fined by the court and sentenced to 6 months in prison – the stiffest penalty available (*Procès de Rose Tamisier*, 1851).

### Catherine Filljung

After the annexation of Alsace-Lorraine by the German Empire in 1871, Catherine Filljung (1848–1915) had visions of the Virgin Mary, who allegedly told her she was on the side of the French people; a highly controversial statement amid tensions between German and French nationalists in the region (Blackbourn, 1993; Klein, 2007). The Bishop of Metz attempted to bring Filljung before the German court for invoking these visions, but the allegations were not sufficient to file a lawsuit, forcing the clergy to change its strategy (Meyer, 1887). In 1884, Filljung had founded a religious orphanage in Biding using donations. Moreover, she was already known in the region for her charismata, including stigmata and miraculous healing. The Diocese of Metz thus accused her of deceiving donors with her ‘fake mysticism’ (Ebel, 1932). Filljung was tried for fraud, spending 4 months in an asylum before the trial. In 1892, the Supreme Court of Leipzig acquitted her, arguing that she suffered from ‘religious madness’ and was not responsible for her actions (cited in Seewann, 2011: 264).

With the above-mentioned cases in mind, the following section explores common legal strategies used to bring alleged mystics before the courts. As I will show below, the two main strategies were to translate mystical phenomena into criminal acts, and to use the ‘demystifying power’ of a lawsuit to ruin the mystic’s reputation.

## Translating mystical phenomena into criminal charges

Criminal courts in Europe were not alone in bringing mystics before the law. Sometimes, the initiative to arrest a mystic came from the Catholic Church itself. Ecclesiastical authorities often saw the enthusiasm surrounding an alleged mystic as a sign of fanaticism. By intervening, they aimed to restrain popular enthusiasm. An ecclesiastical inquiry often preceded and sometimes triggered a judicial investigation. On several occasions, incredulous clergymen refused to examine the supposed mystic. In Filljung’s case, the Bishop of Metz declined to undertake a canonical inquiry for 10 years. Faced with the obstinacy of the diocese, Filljung and the parish priest contacted the Holy Office, who ruled out diabolical intervention but did not confirm that the events were supernatural (Ebel, 1932). A similar conclusion was arrived at for Tamisier. The Archbishopric of Avignon found the phenomena to be extraordinary and unexplained, but denied that they were a ‘true miracle’ (Garçon, 1929: 65). After the Church’s conclusions, the mayor of Saint Saturnin – the French village where the ‘miracles’ took place – wrote a letter to the public prosecutor requesting that he look into the matter:

if, despite all of your investigations, you do not come to know how these things took place, pious people may consider them supernatural. If, on the contrary, you discover a fraud from this girl [Tamisier], I imagine that you will find an article of law with which to punish her. (cited in Garçon, 1929: 72)

As this quotation indicates, transforming mystical phenomena into the subject of criminal charges began by identifying the law allegedly infringed by the defendant. In Tamisier’s case, the problem escalated as it passed through the French juridical system. After several legal confrontations, the assizes declared that the case should fall under Article 262, condemning ‘anyone who, by word or action, shows contempt for the objects of a cult’.^1^ Tamisier’s case was sent to a criminal court, which declared itself ‘incompetent’ to apply Article 262, arguing that Tamisier’s offence pertained to Article 1 (religious defamation). The case landed in the Court of Appeal of Nîmes, which insisted on applying Article 262 and carried out a speedy trial, which found Tamisier guilty (*Procès de Rose Tamisier*, 1851).

Identifying the article of law to be applied depended on establishing the ‘facts’ of the crime. According to Barbara Shapiro (1994: 230): ‘in law, facts involved particulars, that is *singular* events, deeds, or actions’. When brought before the court, alleged mystical phenomena were reduced to actions pointing towards a possible crime, usually fraud. Of course, mystical phenomena are not crimes per se. Transforming such phenomena into actions that were representative of a crime sometimes proved to be a challenge for juridical authorities. Inner mystical experiences could not always be traced through such a transfiguration, especially if there were no outer consequences or objective actions resulting from them. In 19th-century secular courts, accusations focused especially on the physical phenomena of mysticism (see, e.g. Thurston, 1952), not including visions and other internal experiences in criminal cases. It was believed that such bodily phenomena were more objective, because they could be perceived through the senses and thus provide evidence for or against the facts. Thus, they could be more easily examined and expert testimony could be brought to bear on them.

**Figure 2.**
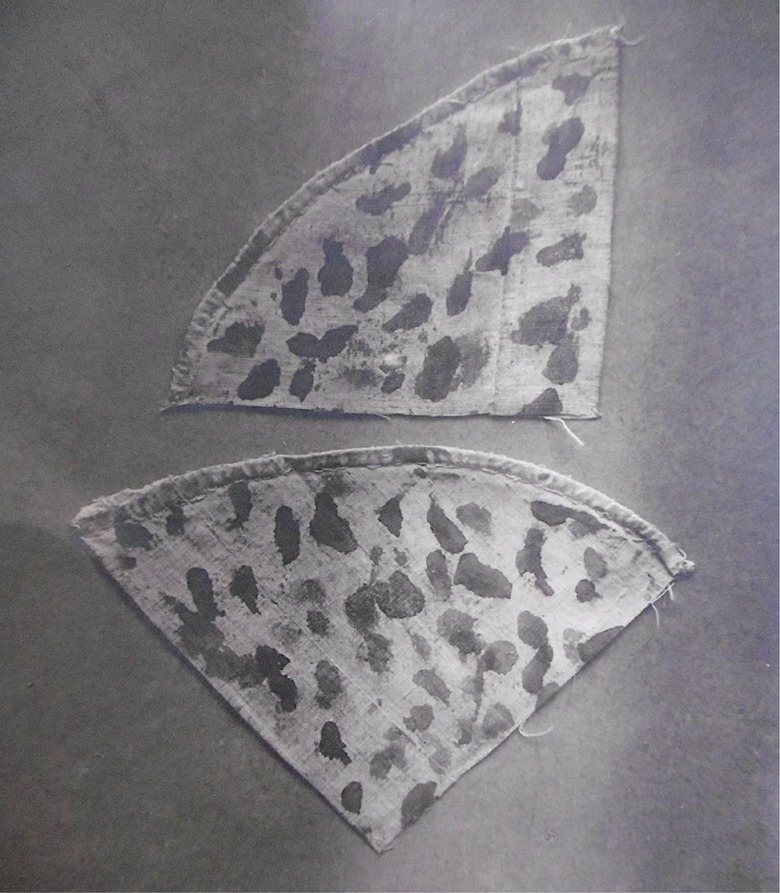
Picture of headbands imprinted with the blood of Filljung’s ‘crown of thorns’ during two Fridays of Lent, 1888 (courtesy of Archives de la Province Dominicaine de France, Bibliothèque du Saulchoir, Paris, Catherine Filljung: VI-Q-62bis).

In the cases considered here, the criminal charges against the defendants referred to only a tiny portion of their mystical experiences. It was their mysticism, however, as a whole that was being judged; I will return to this point later. In order to advance criminal charges against Tamisier, her mystical life was reduced to two actions: having stolen wafers to fake a miraculous Communion and having falsified a religious painting to simulate that it bled (*Procès de Rose Tamisier*, 1851). For her part, Filljung initially faced charges of fraud for faking ecstasy and stigmata (Figure 2), and of misconduct for hiding her own (illicit) children in the orphanage.^2^ In the end, the judge only accepted the accusation of ‘simulation of ecstasies: a criminal means to gain trust’ (cited in Seewann, 2011: 261). Finally, in Sor Patrocinio’s case, the nun was arrested for faking stigmata and trying to subvert the state through political prophecies; since, however, the public prosecutor could find no testimony to support the second accusation, the trial focused on the simulation of stigmata (*Causa formada contra doña María de los Dolores Quiroga*, 1837). In this case, the accusations were not typified into general crimes (e.g. fraud). In other words, the public prosecutor did not even take the trouble to demonstrate that Sor Patrocinio’s actions were against the law (Voltes, 1994).

As we can see, charges concerning mystical phenomena tended to be highly specific, focusing on a single wonder (e.g. stigmata) rather than on the whole range of phenomena that the defendants claimed to experience. The accusers probably believed that proving fraud for one phenomenon would be enough to debunk all other mystical experiences; because a ‘true’ mystic would never fake a supernatural occurrence. In the end, it was therefore the defendants’ mysticism as a whole that was called into question; but what was so disturbing about their mysticism? The specific reasons vary from case to case; what these mystics have in common, however, is that they challenged the ecclesiastical and the political establishments. Filljung founded her religious orphanage without the approval of the bishop; furthermore, she favoured French nationalism in German Alsace-Lorraine (Meyer, 1887). Tamisier’s ‘miracles’ provoked uncontrolled religious enthusiasm during the war of ‘the two Frances’, and rumours linked her to the sect of Eugène Vintras, condemned by the Vatican and the French secular authorities (André, 1851). Finally, Sor Patrocinio’s political prophecies turned her into a symbol of absolutism and a menace to the liberal Spanish regime.

These clashes provoked both ecclesiastical investigations and criminal lawsuits. Unlike canonical inquisition, however, lawsuits had the power to demystify the defendant’s mystical experiences even before a verdict was pronounced. The task of modern criminal justice is not to assess the reality of the supernatural, but to determine whether a criminal act has been committed. Hence, once a lawsuit was initiated, the implicit message was that the alleged mystic was a fraud. After the trials, the defendants never managed to shake off their ‘false mystic’ reputation. They all lost the majority of their supporters at home and abroad. An enraged letter to Filljung, written by a long-standing follower (a woman from Brussels), reveals how what once was admiration could turn into hatred – note that she threatens to have Filljung arrested again, which shows that common people also perceived the law as a legitimate way of pursuing self-styled mystics:

You nasty witch, I urge you to stop bothering me with your letters full of lies… You are certainly not a religious sister, your chapel will never be blessed, everything in you is a lie, you are an audacious rascal; and beware of coming to Brussels, because if I discover you are here, I will have you arrested.

Do not call me your dear friend anymore, because I don’t want to be a friend of Satan, I hate touching the paper on which you write, and do not think that I will give you money to build castles in the air.

Goodbye, my beautiful witch, goodbye…^3^

The demystifying power of lawsuits is one of the reasons why ecclesiastical authorities called upon juridical systems to intervene. Filljung’s case is especially enlightening in this respect. In the opinion of the parish priest, a supporter of Filljung, after the annexation to the German Empire, the Bishop of Metz ‘hoped to find in [Protestant] judges from another cult, born adversaries of the supernatural and, for this reason, their allies and enemies of the defendant’ (Meyer, 1887: 18). Indeed, the Protestant Reformation had redefined the boundaries between the natural and the supernatural and largely rejected post-biblical miracles (Eire, 2016). The parish priest accused the bishop and other clergymen of leaving the case in the hands of German justice. In his opinion, ‘they did not understand the impropriety of bringing before the civil courts the eminently purely religious matters of mysticism and theology’ (Meyer, 1887: 18). Again, the problem relied on defining the nature of what was being judged in order to decide who could judge it. To the parish priest, Filljung’s mystical experiences deserved a proper canonical investigation. To the Bishop of Metz, those experiences were a fraud, and he hoped that the law would punish the self-styled mystic accordingly.

In the meantime, the Bishop of Metz used his own means to repress Filljung and her supporters. Her confessors were excommunicated and Filljung was denied the sacraments (Ebel, 1932). The same punishment was also applied to Tamisier after the assizes found her guilty. To have her situation reversed, Tamisier had to admit her guilt before the Bishop of Avignon, even though she protested her innocence during the trial. After years of resistance, she finally succumbed. The diocesan dossier of her case ends with a statement by which she ‘subjects’ herself, ‘without restriction’, to the conclusions made by the ecclesiastical commission in 1851, which found that Tamisier had not worked any miracles.^4^ Arguably, these pious women suffered more from excommunication from the Catholic Church than from their civil trials. The following section examines the staging of these trials, focusing on two aspects: the public depiction of mystic trials as ‘trials of the supernatural’, and the judicial use of expert testimony to counter such notions and condemn the defendants.

## Mysticism on trial

Mystic trials were rare, but when staged they were a popular event, attracting the curiosity of the national and foreign press. To some extent, the press reported on them as trials of the supernatural. Daily newspapers, such as *La Justice*, chronicled the lawsuit against Filljung under the headline, ‘The misfortunes of a clairvoyant’, and reproduced the court hearings of Tamisier’s case under the title ‘Affair Rosette Tamisier. The miracles of Saint Saturnin’ (see, e.g. ‘Cours et tribunaux’, 1851; ‘Les malheurs’, 1892). As the legal suits escalated, the culture wars of the era crystallized in the journals.

On one side, the Catholic press supported the supernatural to enflame religious enthusiasm against liberal and secularization campaigns. In France, ultramontane newspapers such as *L’Univers* publicized Filljung’s ‘triumphant’ acquittal along with other polemical events related to the Catholic revival, such as the Marian apparitions in Lourdes and La Salette (‘L’Innocence’, 1892; Veuillot, 1860). Similarly, monarchic and Catholic journals in Spain reported on Tamisier’s prodigies and the turmoil they generated in France (see, e.g. ‘Crónica religiosa’, 1851) – and this at a time when neo-Catholicism, a politico-religious ideology that aimed to restore Catholic traditions in the Spanish government, was on the rise (Urigüen, 1986).

**Figure 3.**
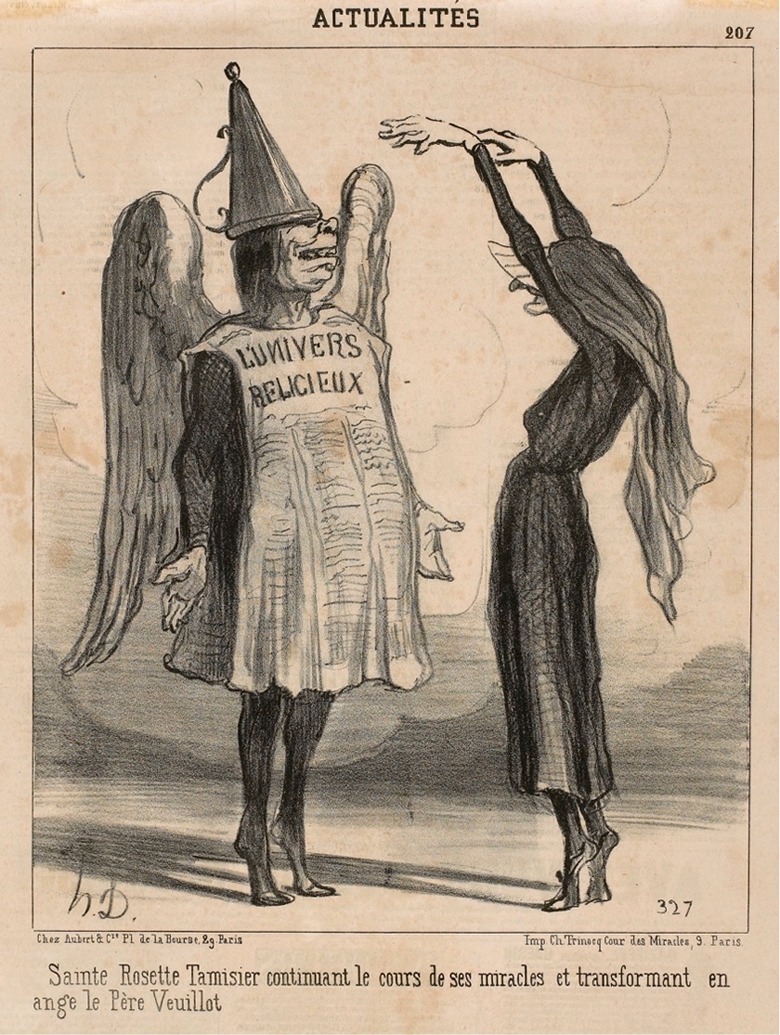
‘Saint Rosette Tamisier continuing with her miracles and transforming Father Veuillot into an angel’. Caricature by Honoré Daumier published in: *Le Charivari*, 18 September 1851.

On the other side, the liberal press used mystic trials to discredit the enemies of the new regimes in Europe. They presented the followers of the defendants as dupes and used the trials to advance their political agendas. In Spain, the liberal press considered those who still believed in Sor Patrocinio’s stigmata to be ‘credulous’ (see, e.g. ‘Causa célebre’, 1836). In France, Louis Veuillot, head of *L’Univers* and an advocate of Tamisier, was ridiculed in cartoons in *Le Charivari* (see Figure 3) and the *Journal pour rire*, led by the Republican caricaturist Charles Philipon (Forbes, 2010). In Germany and Alsace-Lorraine, the press assured its readers that Filljung’s arrest was politically motivated, due to her support of French nationalism after the German annexation. The examining magistrate had to publish a press release to deny this (Ebel, 1932: 221). After Filljung was released, she accused one journal of slander (Seewann, 2011: 213–14).

As mentioned above, the aim of the justice system was not to attest to the reality of supernatural phenomena, but to elucidate the means through which such phenomena could have been feigned. Sometimes, however, judges contributed to the impression that mystic trials were indeed about revealing the truly extraordinary nature of the facts. For example, the stated aim of the criminal case brought against Sor Patrocinio was ‘to discover the origin and provenance of the [holy] wounds…, which were apportioned a supernatural or miraculous character, or such was the aim’ (*Causa formada contra doña María de los Dolores Quiroga*, 1837: inside cover page). During the trial, the lawyers for the defendants had to remind the magistrates that their task was not to make judgements about the supernatural. In his final speech, Tamisier’s defence lawyer warned the judges of the limits of their task:

The supernatural explanation is no more demonstrated than the natural explanation. At this stage, what should be the course of action?

In an ecclesiastical court, I would say: Dismiss the miracle, it is not proved.

But you, the presiding judges, it is not up to you to say if there is a miracle or some trickery, but whether the facts alleged against Rosette Tamisier are proved. (cited in *Procès de Rose Tamisier*, 1851: 19)

Following the common-law tradition, the assessment of facts relied upon the interrogation of witnesses. The credibility of witnesses and their capacity to act as a form of evidence continues to be one of the central issues during trials. Direct testimony (e.g. from eyewitnesses) was sometimes considered sufficient evidence – a belief inherited from the Roman-canon law of proof. Most of the time, however, the judges sought evidence supported by expert testimony; that is, specialists, usually physicians, who had not witnessed the events being examined, but who were asked to give their opinion according to their expertise (Eigen, 1994; Watson, 2006). Expert testimony usually adopted the sceptical ‘devil’s advocate’ attitude in canonization processes. Doctors had been consulted during such processes since at least the 13th-century. In some cases, the expert testimony of physicians had been sufficient to support or repudiate a miracle (Vidal, 2007).

In the pretrial investigations of the cases examined here, the judges called upon expert testimony to examine the claimed mystical phenomena. For example, after her arrest, Sor Patrocinio was removed from her cloistered convent and spent 5 weeks enduring the scrutiny of two surgeons, who cured her ‘holy’ wounds, confirming that they were not supernatural in origin (Jarnés, 1929). In Tamisier’s case, the judge charged a pharmacist with the task of producing a bleeding painting like that of the ‘miracle’ of Saint Saturnin. After many attempts, the pharmacist eventually managed to produce a similar phenomenon using blood from a leech (Garçon, 1929). However interesting, these experiments could not prove that Tamisier or Sor Patrocinio had faked their miracles. Although natural explanations could be used to discredit alleged miracles, they did not constitute criminal evidence while it was not proved that the defendants had committed fraud. Therefore, finding a natural cause of the claimed phenomena was not enough to condemn the defendants in a criminal court; although it would have been enough to debunk such phenomena in an ecclesiastical inquiry.

Why stage such experiments during a trial? In the cases mentioned here, the judges were concerned about the ‘pernicious’ effect that belief in the supernatural had on witnesses, and how it might affect the jury’s verdict. ‘Jurors need evidence’, said the magistrates in Tamisier’s case, ‘but if someone [a witness] claims a supernatural origin [of the facts], who will believe them?’ (Garçon, 1929: 85). More than finding legal proof of the accusations, the experiments mentioned above aimed to debunk the supernatural in the eyes of the public and the jury. In this way, magistrates hoped to stifle popular debate about the authenticity of miracles and mystical phenomena and focus on the crimes of which the defendants were accused.

In 19th-century trials, juridical concerns about the defendant’s intent when committing a crime were of great importance. In Germany, to condemn someone for fraud, lawyers had to prove that the act was committed in ‘bad faith’; that is, with the aim to deceive (Wolffram, 2009). Likewise, in France, jurors had to decide whether the intentions of the defendant were criminal (Harris, 1991). In the case of Tamisier, the public prosecutor expressed the need to ‘appreciate either Rosette Tamisier’s morality, or her macabre habit of simulating miracles’.^5^ During the trial, the jury spokesperson wondered if it would be fair to condemn Tamisier if she admitted having faked the miracle in ‘good faith’ to convert sinners (cited in Garçon, 1929: 85–86). In Spain, the defence lawyer hoped to see Sor Patrocinio acquitted by arguing that the cloistered nun was oppressed and lived without enjoying freedom of speech or action. In both cases, however, the court found that there had been an intention to deceive and declared the defendants guilty (*Causa formada contra doña María de los Dolores Quiroga*, 1837; *Procès de Rose Tamisier,* 1851).

If the defendant was deemed guilty, the alleged miracles and the mystical phenomena were debunked; but what happened if the defendant was acquitted? Although, in juridical terms, it only meant that the defendant was not guilty of the accusations, it also opened the possibility of a supernatural explanation of the phenomena. In 1874, when the mystic Mary Ann Girling (1827–1886) was acquitted of an accusation of lunacy, a crowd of her followers – the ‘Girlingites’ as they were dubbed – cried in jubilation on leaving the courtroom. From that day on, Girling became much more public about her mysticism and began to promote herself to journalists (Popplewell, 1993). This case suggests that judges were concerned about the effect that an acquittal could produce. If alleged mystics were judged and acquitted, enthusiasm for such mystics was likely to increase. Hence, it does not come as a surprise that almost all of the supposed mystics brought before the courts were found guilty. If lawyers could not demonstrate fraud or other charges, declaring the defendant mentally disturbed proved to be effective in stifling religious enthusiasm. In the few cases where the defendants were declared not guilty, the acquittal was supported by a psychiatric diagnosis, usually of hysteria. In this way, the judges were able to conclude that, although the ecstasies and other phenomena may have been real, they were a natural occurrence produced by a mental disorder. The defendant was not a trickster, nor did she have any intention to deceive; but neither was she on a divine mission.

Filljung’s case is the most enlightening in this regard. In 1891, during the pretrial investigation, she was interned for almost 4 months in an asylum in Sarreguemines; that is, more than the maximum of 6 weeks stipulated by the German penal code of 1871 (Wolffram, 2017). The court asked the director of the asylum to examine Filljung’s ecstasies and determine whether there was a deception. A group of psychiatrists performed several tests on her, similar to those practised on other mystics at that time (see, e.g. Guillemain, 2006; Lachapelle, 2004). When the ecstasy began, the doctors pinched Filljung’s flesh and pierced her eyelid to see if the bleeding differed from her ‘blood sweats’.^6^ Building upon ongoing debates concerning hysteria and ‘religious madness’ (see, e.g. Didi-Huberman, 2003; Pires Marques, 2010), the director of the asylum argued before the German court that Filljung was a hysteric and that she was not responsible for her actions. The final sentence concluded that the defendant truly believed in her divine mission, but that her actions were the outcome of a morbid condition that undermined her will. According to German law, because there was no willing intention or ‘bad faith’ to simulate the ecstasies, the defendant was declared not guilty (Pelt, 1934). The medicalization of religious experience allowed Filljung to be acquitted, but at the high price of having her mysticism reduced to a psychiatric diagnosis.

## Conclusions

In an era that witnessed the separation of powers in many European countries, and when modern nation-states aimed to secularize the law, alleged cases of Catholic mysticism were brought before the courts under accusations of fraud, misrepresentation or religious defamation. Such cases took place during times of political turmoil, when the manifestation of the supernatural acquired an ideological meaning. In general, mystic trials had more to do with specific clashes between the defendant and the ecclesiastical or civil authorities than with public concerns about the falsification of mystical phenomena. Filljung’s court case was the result of the hatred of the Bishop of Metz and other clergymen towards her. Sor Patrocinio’s trial was political, disguised by the alleged feigning of stigmata. Tamisier’s lawsuit was inspired by other religious scandals, such as the Vintras’s sect.

To some extent, the cases examined here reveal the aim of the secular powers to control religion amid growing tension within the culture wars in Europe (Clark and Kaiser, 2003). Phenomena that had traditionally been policed by the Church were now regulated inside the courtroom. In Tamisier’s case, the Archbishopric refused to collaborate with the public prosecutor, showing just how much the relationship between Church and state had already deteriorated before the Third Republic. In Germany, Filljung’s acquittal, due to the diagnosis of ‘religious madness’, may be seen as a legacy of the *Kulturkampf*. Finally, in Spain, Sor Patrocinio’s case exemplifies the liberals’ aim to eradicate the hegemonic power of the clergy and detach it, for the first time in Spanish history, from the monarchy.

When confronted with the allegedly supernatural, both ecclesiastical commissions and criminal courts dealt with it in terms of facts. In the courtroom, however, ‘facts’ meant actions related to criminal charges – in these cases, usually fraud. The courts often found it difficult to translate alleged mystical experiences into criminal charges. The chaos experienced during some of the judicial investigations, with judges unable to agree on the right article of law, or concerned about the effect of acquittal with respect to the spreading of a ‘sectarian’ cult, exposes specific concerns raised by mystic trials. During such trials, the followers of the defendant hoped for an acquittal, which they incorrectly interpreted as proof of the supernatural. Acquittals were rare, however, and, when pronounced, they were usually supported by medical reports that cleared the defendant of the accusations but debunked the supernatural with a psychiatric diagnosis.

The coverage these trials received from the national and foreign press, with daily updates and the transcription of the court hearings, reveals the interest they provoked within and beyond national borders. Different narratives coming from Catholic and liberal newspapers show how editors used mystic trials to advance their political agendas. To some extent, the press portrayed such cases as trials of the supernatural. Magistrates, concerned about the pernicious effects of this depiction, attempted to discredit supernaturalist claims through expert testimony. In the 19th century, expert testimony was only required for cases involving serious crimes, such as murder (see, e.g. Eigen, 1994; Harris, 1991). Thus, it is clear that the law deemed the cases examined here to be quite serious, either from the point of view of cultic religious belief or regarding public safety. In the courtroom, medical examinations and scientific experiments served to debunk alleged miracles and mystical phenomena before the jury, the press and the population. Debunking the supernatural, however, rarely amounted to legal proof of the accusations.

Although magistrates perceived the risk of mystic trials encouraging the belief in the supernatural, non-intervention was often deemed more dangerous, especially if the Catholic Church had already failed to restrain enthusiasm surrounding the mystic. For example, in France, the public prosecutor decided to intervene after the Archbishopric of Avignon failed to stop thousands of pilgrims from arriving at the ‘miracle site’ of Saint Saturnin, despite the bishop’s condemnation of Tamisier and ruling out the supernatural.^7^ In such cases, which were rare but very public, trials proved to be an effective tool to discredit alleged mystics. Unlike canonical inquisitions, lawsuits had a certain ‘demystifying power’ that ruined the reputation of the mystics even before the verdict was pronounced. In this vein, the risk of staging such trials proved to be very limited, and their effects, which the defendants deemed ‘calumnious’, continue today. Despite recent attempts by scholars and theologians to vindicate the ‘true mysticism’ of Sor Patrocinio (see the Introduction by Eudaldo Forment in Paredes, 2015) and Filljung (Seewann, 2011), today they are mainly remembered as ‘false mystics’.

## Notes

I wish to thank Fernando Vidal, Tine Van Osselaer, Rhodri Hayward and the anonymous reviewers for their comments.

1. Archives Départementales de Vaucluse (ADV), Avignon, Rosette Tamisier: 13J47.

2. Archives de la Province Dominicaine de France (APDF), Bibliothèque du Saulchoir, Paris, Catherine Filljung: VI-Q-62bis, box 0-12, dossier 1.

3. APDF, VI-Q-62bis, box 0-12, dossier 11, ‘Lettre d’une demoiselle de Bruxelles, 1.12.1894’.

4. ADV, 13J47.

5. ADV, 2Mi732, Letter of the public prosecutor, 1 February 1851.

6. APDF, VI-Q-62bis, box 0-12, dossier 1, ‘cahier Ebel’, p. 255.

7. ADV, 2Mi732; 13J47.
